# Oral cancer treatment: developments in chemotherapy and beyond

**DOI:** 10.1038/sj.bjc.6600591

**Published:** 2002-10-21

**Authors:** V J O'Neill, C J Twelves

**Affiliations:** Cancer Research UK Department of Medical Oncology, University of Glasgow, Alexander Stone Building, Switchback Road, Glasgow G61 1BD, UK

**Keywords:** oral chemotherpay, drug development

## Abstract

Oncology is one of the few areas of medicine where most patients are treated intravenously rather than receiving oral drugs. Recently, several oral anti-cancer drugs have been approved and there are many more in development. Oral chemotherapy is attractive because of its convenience and ease of administration, particularly in the palliative setting. With an increasing number of oral agents emerging, we can expect to see a rapid rise in the use of oral chemotherapy in years to come. This article reviews recent developments in oral chemotherapy, both of traditional cytotoxics and novel, targeted agents, from the viewpoint of patients, physicians, drug developers and health-care providers.

*British Journal of Cancer* (2002) **87**, 933–937. doi:10.1038/sj.bjc.6600591 www.bjcancer.com

© 2002 Cancer Research UK

## 

Oncology is one of the few areas of medicine where most patients are treated intravenously (i.v.) rather than receiving oral drugs. Recently, several oral anti-cancer drugs have been approved and there are many more in development. Oral chemotherapy is attractive because of its convenience and ease of administration, particularly in the palliative setting (Payne, 1992; Liu *et al*, 1997). It is also especially appropriate where prolonged drug exposure is desirable as with schedule dependent agents such as topoisomerase I inhibitors or the fluoropyrimidines. The same argument applies to novel agents such as signal transduction inhibitors and anti-angiogenic drugs that may need to be taken daily for months or years in contrast to the intermittent, short term use of conventional antiproliferative cytotoxics that are often well suited to i.v. administration. With an increasing number of oral agents emerging, we can expect to see a rapid rise in the use of oral chemotherapy in years to come. It is, therefore, timely to consider whether this represents a major change of direction or just a passing fashion in cancer treatment.

More than 20 oral cytotoxics are already available, so why have we remained so reliant on i.v. chemotherapy? One reason is that many oral cytotoxics are new formulations of existing compounds. These are often low-priced ‘generics’ that have not been a high sales priority for the major pharmaceutical companies (Table 1

**Table 1:**
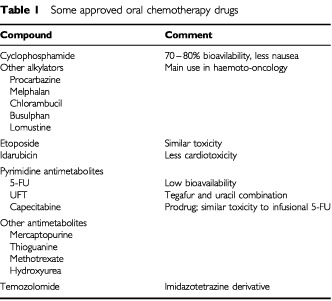
Some approved oral chemotherapy drugs

). For example, cyclophosphamide has been given orally for many years in women with breast cancer as part of some CMF regimens. Oral etoposide is active against a wide range of tumours but enthusiasm for its use has been limited by concerns over toxicity. Currently, probably the widest use of oral chemotherapy is with 6-mercaptopurine, methotrexate and busulphan in patients with leukaemia and lymphoma. A second factor limiting the use of newer oral chemotherapy agents has been the narrow clinical setting in which their use is indicated. This has limited the impact of drugs such as temozolamide (approved for glioma, astrocytoma and melanoma only in the UK) and idarubicin (principally used in leukaemia). Similarly, capecitabine was initially licenced only for use in women with advanced breast cancer that was resistant to taxanes. Another factor, more difficult to define, is perhaps that amongst both clinicians and drug developers there has been an implicit assumption that anticancer drugs are best given i.v.

## CYTOTOXICS

Over the last decade oral chemotherapy has generally failed to keep pace with increasing use of i.v. cytotoxics. Japan is an exception where there has been widespread use of oral chemotherapy, especially in patients with gastrointestinal cancers, during the mid-1990s. Elsewhere, however, the continued dominance of i.v. treatment reflects in part the range of new drugs and also novel classes of agent that have been available for i.v. use.

Many emerging oral cytotoxics are new formulations of drugs routinely given i.v. (Table 2

**Table 2:**
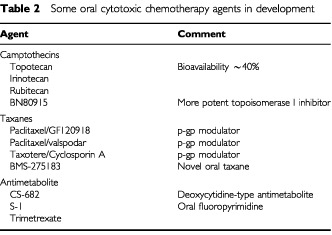
Some oral cytotoxic chemotherapy agents in development

). Topotecan is equally effective in small cell lung cancer by the i.v. and oral routes but oral treatment has a better toxicity profile with less myelosuppression (von Pawel *et al*, 2001). The ability to administer taxanes orally would offer considerable advantages, particularly with paclitaxel where the chremophore EL vehicle can be responsible for hypersensitivity reactions. Coadministration of cyclosporin A, a potent inhibitor of p-glycoprotein, results in oral bioavailability for taxanes in excess of 50%. Several oral taxanes are also in development (Polizzi *et al*, 2000; Rose *et al*, 2001) and likely to become available in the next few years.

Because 5-fluorouracil (5-FU) is highly schedule dependent, but oral administration is unreliable and causes diarrhoea, this has been a particular focus of efforts to develop oral alternatives. These have included pro-drugs that are absorbed unchanged (capecitabine, tegafur), addition of inhibitors of the enzyme DPD that catabolises 5-FU (uracil, eniluracil), or a combination of the two (UFT, S1, emitefur) (reviewed by Lamont and Schilsky, 1999). Despite the elegance of these approaches, their development has not been straightforward. Eniluracil is a very effective DPD inhibitor that renders 5-FU highly bioavailable after oral administration but development has been suspended on the basis of disappointing results in phase III trials. The combination of UFT and folinic acid is active in fluoropyrimidine sensitive tumours but has not been approved for use in the US with doubts regarding its efficacy in relation to i.v. 5-FU remaining. The future of S1 and emitefur is also unclear. The exception is capecitabine which is now approved across the world in two common solid tumours, breast and colorectal cancer. Capecitabine is as effective as the ‘Mayo Clinic’ i.v. regimen of 5-FU in metastatic colorectal cancer, achieving higher response rates with equal time to progression and overall survival. It is also active as a single agent in taxane resistant breast cancer. Results of a recent trial in patients with anthracycline resistant breast cancer show that the addition of capecitabine to docetaxal significantly prolongs survival (O'Shaughnessy *et al*, 2002). The success of capecitabine means that the profile of oral chemotherapy is set to rise.

## NOVEL AGENTS

Potentially even more important in the medium and long-term will be ‘smart’ drugs, targeted to components of intra-cellular signalling pathways such as protein kinases (Rajagonopalan *et al*, 2000). Several are being developed as oral therapies with imatinib and iressa leading the way (Table 3

**Table 3:**
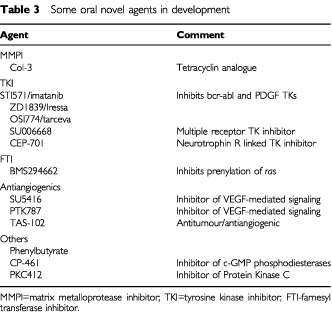
Some oral novel agents in development

). Imatinib inhibits the c-*abl* and c-*kit* tyrosine kinases. It has excellent oral bioavailability and remarkable activity in both CML (Kantarjian and Talpaz, 2001) and G.I. stromal tumours (van Oosterom *et al*, 2001), which are ‘driven’ by the bcr-able fusion protein and c-*kit*, respectively. Iressa (ZD 1839) is an oral anilinoquinazoline inhibitor of EGFR tyrosine kinase activity given once daily by mouth that is active in non-small cell lung cancer (Baselga *et al*, 2000). A reversible acneiform rash has been observed with both iressa and tarceva (OSI-774), another oral quinazoline EGFR tyrosine kinase inhibitor also active in non-small cell lung and head and neck cancers (Perez-Soler *et al*, 2001; Senzer *et al*, 2001). The same rash is seen with cetuximab, an i.v. monoclonal antibody targeted at the EGFR receptor (Baselga, 2001). This overlap in toxicities between oral and i.v. compounds with distinct molecular characteristics emphasises that route of administration alone will not necessarily avoid a ‘class effect’ toxicity.

## PERSPECTIVES ON THE DEVELOPMENT OF ORAL ANTI-CANCER DRUGS

Notwithstanding these developments, the future of oral anti-cancer treatments is not entirely clear. Concerns remain regarding compliance, absorption and variable pharmacokinetics as well as re-imbursement in some countries. Perspectives on the use of oral chemotherapy differ between the patient, the oncologist, the pharmaceutical industry and the funders of health care.

### The patient

A clear majority (>80%) of patients prefer oral chemotherapy, but only provided this is not at the expense of efficacy (Liu *et al*, 1997; Borner *et al*, 2002). The preference for oral chemotherapy stems largely from the greater convenience of treatment at home (cited by 57% of patients) and avoidance of venepunctures (55%). There are additional concerns with protracted infusional regimens where many patients experience problems with indwelling venous catheters. A third of patients also cited the greater sense of ‘control’ over their treatment that they would gain from oral chemotherapy (Liu *et al*, 1997). Quality of life is especially important in the palliative setting and oral treatment can reduce the disruption of home life for both the patient and their family (Payne, 1992; De Mario and Ratain, 1998). Similar considerations apply in the adjuvant setting where patients are seeking to resume their normal lifestyle following diagnosis and primary treatment.

Taking treatment at home over a prolonged period does, however, present challenges. It is often said that for some patients reduced contact with the oncology team may be a disadvantage if it limits the support they receive, although there is little evidence to support this assertion. More easy to define is the importance of educating patients. Experience with capecitabine has shown it is essential that patients recognise side-effects so that treatment can be interrupted, preventing these toxicities becoming more serious (Cassidy *et al*, 1999). Education of the patient, as well as their G.P., practice nurse and pharmacist, will be of crucial importance where anticancer drugs are being taken primarily in the community rather than at the hospital.

### The oncologist

From the medical perspective, oral chemotherapy requires us to look at many of our preconceptions about cancer treatment. Like our patients, as oncologists we want oral treatment to be as effective as i.v. therapy with no increase in toxicity, especially vomiting and diarrhoea. Oncologists recognise that in breast cancer responses to endocrine therapy are longer lasting and achieved with less toxicity than with chemotherapy, but there is often a prejudice that in other settings i.v. treatment is inherently more reliable and more effective. A more substantive concern is that oral drugs may have low and unpredictable bioavailability in contrast to the immediate and complete bioavailability after i.v. administration. Variable oral bioavailability is likely to be a function of variable degradation in gastrointestinal fluids, intestinal P-glycoprotein and interaction with intestinal and liver cytochrome P450 (CYP3A4) catalytic activity which varies as much as 10-fold among individuals (Guengerich, 1992). In addition, some drugs, such as etoposide, exhibit saturable absorption so bioavailability is much lower at high doses. This does not limit the use of oral etoposide at standard doses, but does complicate the widespread of oral leucovorin, which demonstrates only 31% bioavailability at a 200 mg dose.

This concern regarding bioavailability is understandable for a class of drugs with a narrow therapeutic index, but is perhaps exaggerated in relation to the evidence. Despite direct intravascular delivery, the pharmacokinetics of i.v. cytotoxics vary widely. Indeed, when the pharmacokinetics of i.v. and oral idarubicin were compared, there was substantial variability in kinetics using both routes of administration. Improved methods of formulation and drug delivery along with fractionated dosing should overcome many of these pharmacokinetic problems.

Compliance is a major consideration in any large scale shift to oral anticancer treatment. These concerns are usually voiced by oncologists rather than their patients. Reports on compliance are, however, conflicting. Intuitively, and in view of the seriousness of their condition, patients with cancer might be thought a group unlikely to default on treatment. High rates of compliance were shown using an innovative electronic tablet bottle in patients with lymphoma, small cell lung cancer and ovarian cancer patients (Lee *et al*, 1992, 1993, 1996). On the other hand, a surprising 43% of patients receiving oral cyclophosphamide as part of treatment for breast cancer did not take their medication as prescribed (Lebovits *et al*, 1990). Interestingly, in this study some patients took more than the prescribed dose. This phenomenon of ‘over-compliance’ may be a concern if patients mistake their dose or continue treatment in the face of side-effects.

Many factors impact on treatment compliance but their importance in patients with cancer is unclear. In general, compliance is higher in older, better educated, more affluent patients living in smaller family groups. Compliance is also improved by limiting the number of tablets (a maximum of 6–8 per day has been suggested), making them easy to swallow and using a simple dosing schedule. By contrast, compliance tends to fall with chronic administration, which may be an issue with signal transduction inhibitors and other novel approaches. Education of the patient and their family, daily blister packs and frequent contact with the prescribing centre through weekly telephone calls from a nurse specialist, should reduce non-compliance, but supportive evidence is lacking.

With patients taking their treatment at home rather than at the hospital, communication with health care workers in the community will be increasingly important. It is, however, important that trained oncologists continue to prescribe and monitor the use of oral anticancer drugs. We must remember the lesson of oral etoposide which caused serious toxicity; anti-proliferative cytotoxics are potentially toxic irrespective of the route of administration and must be carefully supervised. Oral agents with novel modes of action may also have unexpected side-effects, especially as the optimum duration of treatment is unclear and patients may remain on treatment for many years. It may also not be clear how and when to modify doses. The appropriate use of drugs targeted at specific signalling pathways may require that each patient's tumour must undergo molecular characterisation in the oncology centre. All of these issues require that the prescription and monitoring of oral chemotherapy be restricted to the oncologist.

### The pharmaceutical industry

Oral chemotherapy will only become available if new agents are developed and promoted by the pharmaceutical industry. From their perspective oral formulations may be more expensive to develop with issues such as the effect of food on absorbtion and drug interactions more of a concern than with their intravenous counterparts. Oral formulations will not be appropriate or feasible for all drugs. There is, for example, little to be gained clinically from developing an oral form of a conventional antiproliferative that can be given simply once every 3 weeks as a bolus i.v. injection. An early decision to develop oral compounds can be made if their mode of action makes it clear that prolonged daily dosing will be required.

The development of oral chemotherapy is not straightforward. Some patient populations such as those who have undergone upper G.I. tract surgery, those with head and neck cancer and children may not be good candidates for oral treatment. Compliance may also be a concern during early trials if apparent ‘tolerance’ is due to non compliance which could render identification of a safe dose difficult. Other dosing issues are also different for oral drugs and the argument for ‘flat dosing’ irrespective of body surface area, is especially strong for oral drugs. With many i.v. cytotoxics there is little or no evidence to support adjusting doses for body surface area, but the dose rounding that is a consequence of having a limited number of fixed table dosages will require a change in attitude from those developing and prescribing anti-cancer drugs.

All these factors indicate that the decision to pursue development of an oral compound points to the need for multiple, well designed trials and population pharmacokinetic studies. The development of an oral anti-cancer drug is likely to require more innovation and may be more expensive than traditional i.v. drug development, but many of these challenges have been overcome in other therapeutic areas. The rewards for the successful development of effective, novel oral anti-cancer drugs are potentially immense.

### The health-care system

Funding of health-care is an issue everywhere, although the specific concerns vary from country to country. Assuming equivalent efficacy, we can expect that patients would choose oral chemotherapy but will physicians be able and willing to follow their lead? A particular problem is that in some countries oral treatment may generate less income for the hospital and the doctor.

Oral treatment will reduce the number of in-patient and out-patient hospital visits with their associated medical and nursing administrative costs, avoid the expense of disposables (e.g. infusion equipment, pumps) and decrease the pharmacy workload (Twelves *et al*, 2001). Currently, chemotherapy costs account for only a small proportion of the direct cost of cancer care so it should be possible to set increased drug costs against the substantial savings that will be made elsewhere. Drug budgets are, however, easily identified and this process will be easier in some countries than in others.

Differences in methods of reimbursement and physician remuneration between Europe and the United States mean that American oncologists may be less enthusiastic about oral chemotherapy. Medicare has generally not reimbursed costs of oral chemotherapy, except where the oral formulation is a form of the i.v. drug. This has had clear implications for the use of oral chemotherapy. In the world's major market the recent agreement by Medicare to reimburse the cost of capecitabine in the palliative setting signals an important shift in attitude. An interesting analogy can be drawn with the use of oral as opposed to intravenous gancyclovir in the treatment of CMV retinitis. Although the oral drug costs were substantially higher, overall a significant cost differential emerged in favour of oral therapy in terms of administrating treatment, monitoring the patient and managing adverse events (Sullivan *et al*, 1996). The net result is that oral gancyclovir therapy increases revenues for the manufacturer but reduces revenues for the profit-making health care providers. If the same is true for oral chemotherapy there will be a substantial impact on the provision of health care.

## CONCLUSIONS

The outlook for oral chemotherapy is positive. Having been for many years the poor relation, oral chemotherapy is poised to become a major force. Encouraging clinical trial results indicate that for the first time many of the oral anti-cancer drugs in development are actually better drugs rather than pale imitations of i.v. treatments. Many of these new agents, especially signal transduction inhibitors or angiogenesis inhibitors, will be available only as oral treatments with an i.v. dosage form either impractical or inappropriate.

In an era when patients are increasingly well informed and consumerism plays a more prominent role in health care, patients' preference for oral treatment is a powerful force. Both the patients and their doctors will, however, only embrace oral chemotherapy so long as it is as effective as i.v. treatment. For the pharmaceutical industry substantial hurdles remain in the development of oral anti-cancer drugs. Nevertheless, the large pharmaceutical companies are investing heavily in oral drug development. When approved, these oral agents will receive correspondingly large marketing support building on the preference of patients for oral treatment. This will present doctors and others involved in delivering anti-cancer treatment with challenges in terms of re-organising structures to deliver oral treatment.

Not all new agents will be oral and there are many exciting novel compounds that are given i.v. Intravenous administration will remain an important route of delivery, but we can expect oral chemotherapy to have an increasing impact over the next years. In the longer term, oral chemotherapy may well be the rule rather than the exception.
